# Repeatability and Reproducibility of Measures of Bovine Methane Emissions Recorded using a Laser Detector

**DOI:** 10.3390/ani10040606

**Published:** 2020-04-01

**Authors:** Giovanni Niero, Filippo Cendron, Mauro Penasa, Massimo De Marchi, Giulio Cozzi, Martino Cassandro

**Affiliations:** 1 Department of Agronomy, Food, Natural resources, Animals and Environment, University of Padova, Viale dell’Università 16, 35020 Legnaro (PD), Italy; filippo.cendron@phd.unipd.it (F.C.); mauro.penasa@unipd.it (M.P.); massimo.demarchi@unipd.it (M.D.M.); martino.cassandro@unipd.it (M.C.); 2 Department of Animal Medicine, Production and Health, University of Padova, Viale dell’Università 16, 35020 Legnaro (PD), Italy; giulio.cozzi@unipd.it

**Keywords:** methane emission, environment, sustainability, efficiency

## Abstract

**Simple Summary:**

The collection of phenotypes related to livestock methane emissions is hampered by costly and time-demanding techniques. In the present research, a laser methane detector was used to measure several novel phenotypes, including mean and aggregate of methane records, and mean and number of methane peak records, considering Simmental heifers as a case study. Phenotypes showed satisfactory repeatability and reproducibility for log-transformed data. The number of emission peaks had great variability across animals and thus it is a promising candidate to discriminate between high and low emitters.

**Abstract:**

Methane (CH_4_) emissions represent a worldwide problem due to their direct involvement in atmospheric warming and climate change. Ruminants are among the major players in the global scenario of CH_4_ emissions, and CH_4_ emissions are a problem for feed efficiency since enteric CH_4_ is eructed to the detriment of milk and meat production. The collection of CH_4_ phenotypes at the population level is still hampered by costly and time-demanding techniques. In the present study, a laser methane detector was used to assess repeatability and reproducibility of CH_4_ phenotypes, including mean and aggregate of CH_4_ records, slope of the linear equation modelling the aggregate function, and mean and number of CH_4_ peak records. Five repeated measurements were performed in a commercial farm on three Simmental heifers, and the same protocol was repeated over a period of three days. Methane emission phenotypes expressed as parts per million per linear meter (ppm × m) were not normally distributed and, thus, they were log-transformed to reach normality. Repeatability and reproducibility were calculated as the relative standard deviation of five measurements within the same day and 15 measurements across three days, respectively. All phenotypes showed higher repeatability and reproducibility for log-transformed data compared with data expressed as ppm × m. The linear equation modelling the aggregate function highlighted a very high coefficient of determination (≥0.99), which suggests that daily CH_4_ emissions might be derived using this approach. The number of CH_4_ peaks resulted as particularly diverse across animals and therefore it is a potential candidate to discriminate between high and low emitting animals. Results of this study suggest that laser methane detector is a promising tool to measure bovine CH_4_ emissions in field conditions.

## 1. Introduction

Greenhouse gases (GHG) represent a worldwide problem for their impact on global warming and climate change. Anthropic activities related to the primary and secondary sectors are responsible for the majority of GHG released in the atmosphere. Methane (CH_4_) is considered one of the most detrimental GHG, with a global warming potential 28-fold greater than that of carbon dioxide [1]. The livestock sector is estimated to account for 18% of global anthropogenic GHG emissions, and ruminants are the main emitters of atmospheric CH_4_ [2]. Ruminants produce from 250 to 500 L of CH_4_ per head per day and are estimated to account for 8% to 10% of global warming in the next century [3]. Besides environmental and ecological issues, CH_4_ emissions in ruminants represent a problem in terms of feed and production efficiency, since 2% to 12% of cattle gross energy intake is lost through CH_4_ eructation [4].

For decades, the scientific community has focused on different strategies and approaches aiming at reducing ruminants CH_4_ emissions, which, at least partially, succeeded in lowering environmental impact and increasing production efficiency at the same time. Nevertheless, large-scale collection of individual phenotypes related to CH_4_ emissions is still hampered by high recording costs and time-demanding techniques, including respiration chambers, GreenFeed (C-Lock Inc., Rapid City, SD, USA), and sniffer-based systems [5]. For these reasons, the development of alternative, cost-effective, and rapid tools for CH_4_ measurement or prediction is an emerging research field of global interest. Until now, mid-infrared spectroscopy [6], archaeol quantification [7], sulphur hexafluoride tracer, and CO_2_ to CH_4_ ratio [8] have been investigated as indirect proxies for CH_4_ production in cattle. Laser methane detector (LMD) has been proposed as an alternative instrument to directly measure CH_4_ emissions. The sensitivity and accuracy of LMD have been assessed in controlled conditions, by comparing data acquired through LMD with those measured through the respiration chamber (coefficient of determination, R^2^ from 0.64 to 0.97) [9,10] and GreenFeed (R^2^ = 0.64) [11]. However, it is still unclear if LMD may be appropriate in terms of repeatability and reproducibility to accurately measure CH_4_ emissions in field conditions. 

This research question was investigated by assessing repeatability and reproducibility of different CH_4_-related phenotypes, including mean and aggregate of CH_4_ records, slope of the linear equation modelling the aggregate function, and mean and number of CH_4_ peak records. For this purpose, CH_4_ emissions were measured on Simmental heifers as a case study.

## 2. Materials and Methods

### 2.1. Experimental Design and Measurements of CH_4_

Procedures used in this study are excluded from the authorization of the animal welfare committee. Methane emission measurements were performed in September 2019, in a commercial dairy farm located in Padova province (north-east of Italy) on three pregnant Simmental heifers: heifer 1 (650 kg live weight, 22.6 months of age), heifer 2 (530 kg live weight, 17.3 months of age), and heifer 3 (440 kg live weight, 16.7 months of age). Animals were housed in the same open-aerated barn. Heifers received the same diet containing wheat straw (24.1%, w/w), corn silage (17.5%, w/w), meadow hay (15.3%, w/w), protein mix (9.6%, w/w), corn meal (2.2%, w/w), and mineral/vitamin mix (0.7%, w/w), distributed through a total mixed ration. Protein, neutral detergent fiber, acid detergent fiber, starch, fat, and ashes content, calculated on dry matter basis, were 12.39%, 57.12%, 36.25%, 5.30%, 2.62%, and 7.32%, respectively.

**Figure 1:**
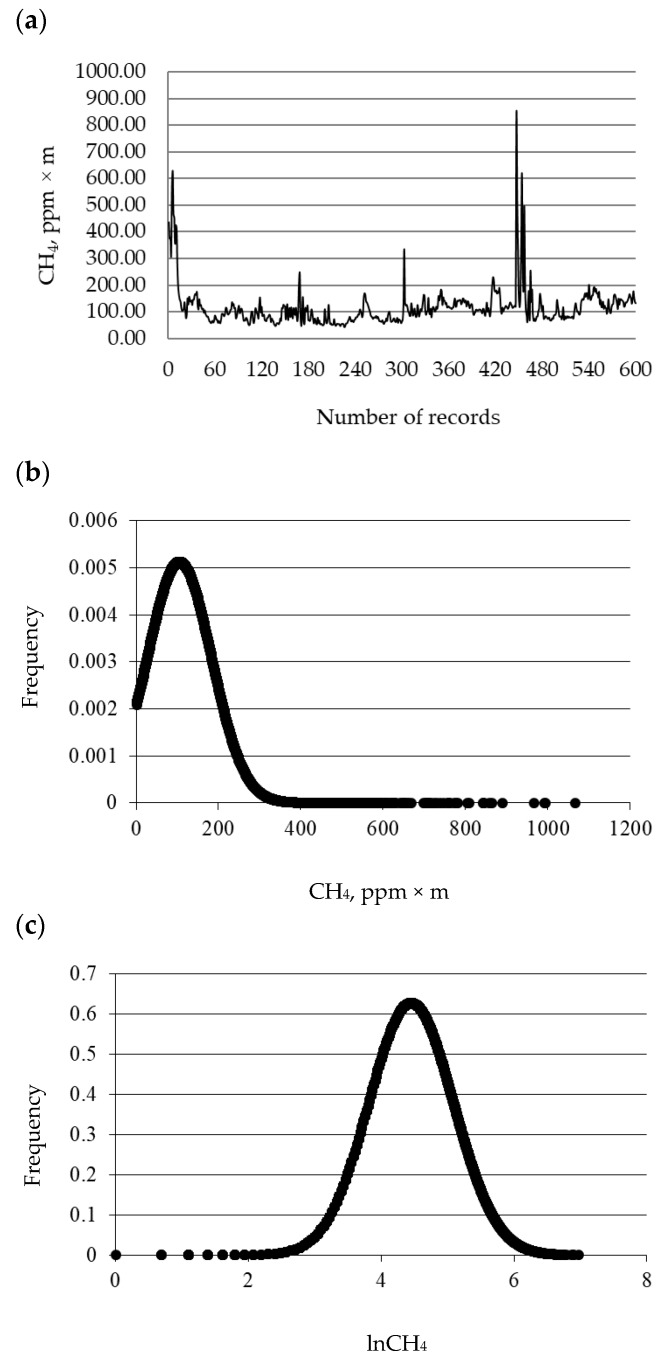
(**a**) Methanogram plot representing records (n = 600) of CH_4_ emissions (ppm × m) collected during one measurement of a heifer, (**b**) distribution of CH_4_ emissions (n = 27,000) expressed as ppm × m, and (**c**) distribution of CH_4_ emissions (n = 27,000) expressed as log_e_-transformed data (lnCH_4_).

Methane emissions were measured through Laser Methane Mini (Crowcon, Abingdon, UK) and CH_4_ was expressed as parts per million per linear meter (ppm × m). Each measurement was performed by pointing the laser to the nostril of a single animal for 5 min, at a distance of 3 m, according to the protocol proposed by Chagunda et al. [12]. Each heifer was restrained in a single pen but it could perform natural activities such as standing, lying, eating, and ruminating. The LMD was set to detect one record of CH_4_ emission every 0.5 s, for a total of 600 records for each measurement (5 min). Every single measurement was forwarded via Bluetooth from the laser device to a Lenovo Tab E7 (Lenovo, Hong Kong, China) tablet, equipped with an Android operating system and Gas Viewer (Tokyo Gas Engineering, Tokyo, Japan) application, saved as .csv file, sent to a dedicated e-mail box, and downloaded in a computer workstation to allow for the local storage of data. The methanogram plot resulting from a single measurement is depicted in Figure 1a. Five consecutive measurements were performed within a day for each animal, which achieved a total of 15 measurements and 9000 records per day. All measurements were performed at the same time of the day (between 8:00 a.m. and 9:30 a.m.), and in the same order for the three animals involved in the study. The protocol was repeated for three consecutive days. As a result, 15 measurements were performed across three days for each heifer, achieving a final dataset of 45 measurements and 27,000 records.

### 2.2. Data Editing

The distribution of CH_4_ emissions expressed as ppm × m is depicted in Figure 1b. Records exceeding three standard deviations from the mean were discarded from the original raw dataset (ppm × m, n = 27,000), which led to 26,449 records available for subsequent analysis. The original raw data of CH_4_ emissions were log_e_-transformed (lnCH_4_) to achieve normality and homogeneity of variances. The distribution of the probability function of lnCH_4_ is reported in Figure 1c. Additionally, in this case, records exceeding three standard deviations from the mean were deleted from the original dataset (lnCH_4_, n = 27,000), which resulted in 26,856 records available for subsequent analysis.

### 2.3. Repeatability and Reproducibility of Phenotypes

Phenotypes considered in the present study were: (i) mean of CH_4_ and lnCH_4_ records, (ii) aggregate of CH_4_ and lnCH_4_ records, (iii) slope of the linear equation modelling the aggregate function, (iv) mean of CH_4_ and lnCH_4_ peak records, calculated on the last decile of the distribution, and (v) number of CH_4_ and lnCH_4_ peak records. Repeatability of the previously mentioned phenotypes was calculated as the relative standard deviation (RSD_r_) of five consecutive measurements carried out within the same day and within the same animal. Similarly, reproducibility of phenotypes was calculated as the relative standard deviation (RSD_R_) of 15 measurements collected across three days of analyses and within the same animal, as proposed by Chagunda et al. [12], Franzoi et al. [13], and Niero et al. [14].

## 3. Results and Discussion

### 3.1. Distribution and Descriptive Statistics

Figure 1a depicts the methanogram plot of 600 records of CH_4_ emissions expressed as ppm × m, obtained in a single measurement on a single heifer. The methanogram featured a baseline signal, including the majority of records, which is likely due to environmental CH_4_ and to the basal eructation activity. The plot highlighted clear emission signals, as the minority of records, associated with peaks of CH_4_ eructation. Methane emissions averaged 105.48 and 98.26 ppm × m with standard deviation of 77.92 and 58.02 ppm × m for the pre-edited and post-edited datasets, respectively (Table 1). Average CH_4_ emissions of the present study was about half the mean value reported by Chagunda et al. [12]. In terms of variability, the standard deviation in our study was lower than that obtained by Chagunda et al. [12]. These differences are likely due to the different experimental design adopted by Chagunda et al. [12] who measured CH_4_ in lactating cows. Methane emissions in the present study were lower even when compared with Sorg et al. [10], and this difference is likely due to (i) the diverse conditions of measurements, (ii) different categories of animals, and (iii) the different feed administered to animals in the two studies. In the present study, measurements were carried out on Simmental heifers housed in an aerated barn, whereas Sorg et al. [10] measured CH_4_ exhaled from Holstein Friesian lactating cows in the spent air of the respiration chamber. Overall mean, mode, and median of CH_4_ expressed as ppm × m were rather different from each other and thus, skewness and kurtosis were relatively far from zero (Table 1). Visual inspection of data distribution (Figure 1) and Shapiro Wilk’s test suggested that CH_4_ expressed as ppm × m was not normally distributed (*p* < 0.05). The log_e_-transformation of CH_4_ produced a much more normal trait (lnCH_4_), as reported in Figure 1. Methane emissions averaged 4.45 and 4.46 in the pre-edited and post-edited dataset, respectively. Mode and median were 4.16 and 4.48, and Shapiro Wilk’s test was not statistically significant (*p* > 0.05), both in the pre-edited and in the post-edited datasets. Skewness and kurtosis were close to zero in the pre-edited and post-edited dataset. Logarithmic transformations were proposed also by Ali and Shook [15] and Benedet et al. [16] to achieve normal distributions and homogeneity of variances for the milk somatic cell count and blood β-hydroxybutyrate, respectively.

### 3.2. Mean and Aggregate of CH_4_ Emissions

The precision of LMD for determining the mean of CH_4_ emissions was assessed through RSD_r_ and RSD_R_ (Table 2). Methane emissions expressed as ppm × m showed poor repeatability and reproducibility. Repeatability was always greater than 40%, with the minimum value reported for heifer 1 in day 3 (41.57%), and the maximum (74.48%) for heifers 3 and 2 in day 1 and 3, respectively. Reproducibility mirrored the same great variability, ranging from 52.43% (heifer 1) to 56.03% (heifer 2). On the other hand, lnCH_4_ highlighted notable improvements in terms of RSD_r_ and RSD_R_. Repeatability varied from 8.93% (heifer 1 in day 3) to 14.85% (heifer 1 in day 1), whereas reproducibility ranged from 11.98% (heifer 2) to 15.35% (heifer 3). Still, such repeatability and reproducibility values remain greater than values from other studies describing the precision of analytical methods carried out under controlled conditions [13,17]. Overall, the variability observed in the present study was likely due to (i) different physiological activities and behaviours of the animals throughout measurements (e.g., eating, standing, and ruminating) [12], and (ii) the environmental factors inherent to the on-field approach, with particular regard to temperature, wind velocity, proximity of other animals, and humidity [5].

The aggregate value of CH_4_ records, the slope, and the coefficient of determination (R^2^) of the linear equation modelling the aggregate function are reported in Table 3. Aggregate values of CH_4_ emissions showed the lowest value for heifer 3 in day 1, being equal to 153,393 ppm × m and 11,212 lnCH_4_. The greatest aggregate value was observed for heifer 1 in day 2 (363,110 ppm × m and 14,313 lnCH_4_). The slopes of the linear equation mirrored the tendency of aggregate values, being lower and greater concurrently with lower and greater aggregates. Although the agreement between aggregates and slopes may support and reinforce the significance of these traits, it can be argued that the consideration of both phenotypes is redundant since their biological meaning is likely the same. Overall, the R^2^ of the aggregate functions of lnCH_4_ (0.999) was greater than the R^2^ of the aggregate functions of CH_4_ (0.989 to 0.997). Such a great accuracy suggests that the linear equation modelling the aggregate function might be used in the future to estimate long-term or daily CH_4_ emissions.

### 3.3. Peaks of CH_4_ Emissions

The precision of LMD for determining peaks of CH_4_ and lnCH_4_ emissions was assessed through RSD_r_ and RSD_R_ (Table 4). Repeatability and reproducibility for peaks of CH_4_ emissions showed greater precision compared with the same indexes calculated for means of CH_4_ emissions. This translated into a relatively low RSD_r_, ranging from 17.58% to 20.15% for CH_4_, and 4.50% to 5.34% for lnCH_4_, and RSD_R_, which was always lower than 20% and 5% for CH_4_ and lnCH_4_, respectively. The average values for peaks of CH_4_ emissions did not vary much across different animals, which suggests that this phenotype might be not adequate to discriminate between high and low CH_4_ emitters. Such a low variability was somewhat expected. Peaks of CH_4_ emissions were defined as records belonging to the highest decile of both the datasets, which leads to a considerable decrease of variability. For this reason, the number of peaks emitted from every single animal is more informative because it is more differentiated across heifers. A similar approach was adopted by Bobbo et al. [18] in the study of new selection tools for mastitis resistance in dairy cows assuming different alternative somatic cell count traits as indicators of the mastitis event.

## 4. Conclusions

The present research assessed the repeatability and the reproducibility of phenotypes related to CH_4_ emissions, measured through LMD using Simmental heifers as a key study. The distribution of emission events expressed as ppm × m showed a significant deviation from the normal distribution, but the logarithmic transformation of the data led to normality. Repeatability and reproducibility were much better for lnCH_4_ than for CH_4_. The coefficient of determination of the linear equation modelling the aggregate function showed high precision. Such results are promising since these equations might be used to estimate daily or long-term CH_4_ emissions. Peaks of CH_4_ emissions were rather different across animals in terms of the number of events but were homogeneous in terms of average values. For this reason, the number of peaks may be an interesting phenotype to discriminate between high and low emitting animals. Overall, results of the present study indicate that measures carried out through LMD are fairly repeatable and reproducible. Therefore, in terms of accuracy, LMD may be considered as a promising tool enabling to measure bovine CH_4_ emissions in field conditions at relatively low costs. Future studies will focus on the application of LMD for large-scale studies to assess sources of variation of CH_4_ emissions.

## Figures and Tables

**Table 1:** Descriptive statistics of methane (CH_4_) and log_e_-transformed methane (lnCH_4_) emissions.

Item	n	Mean	Mode	Median	Skewness	Kurtosis	Minimum	Maximum	SD ^1^
CH_4_, ppm × m									
Pre-editing	27,000	105.48	64	88	2.92	14.73	1.00	1067.00	77.92
Post-editing	26,449	98.26	64	86	1.36	2.18	1.00	339.00	58.02
lnCH_4_									
Pre-editing	27,000	4.45	4.16	4.48	−0.08	0.98	0.00	6.97	0.64
Post-editing	26,856	4.46	4.16	4.48	0.06	0.08	2.57	6.37	0.61

^1^ SD, standard deviation.

**Table 2:** Number of records, mean, repeatability relative standard deviation (RSD_r_, %) calculated within the day on five measurements, and reproducibility relative standard deviation (RSD_R_, %) calculated across days on 15 measurements for methane (CH_4_) and log_e_-transformed methane (lnCH_4_) emissions.

Item	Day 1			Day 2			Day 3			Overall		
	Records	Mean	RSD_r_	Records	Mean	RSD_r_	Records	Mean	RSD_r_	Records	Mean	RSD_R_
CH_4_, ppm × m												
Heifer 1	2941	84.61	67.03	2897	125.34	45.48	2913	123.76	41.57	8751	111.12	52.43
Heifer 2	2943	95.76	57.31	2925	97.90	57.23	2945	51.34	74.48	8813	104.07	56.03
Heifer 3	2988	51.33	74.48	2946	110.40	50.23	2951	78.14	67.84	8885	79.81	55.01
Overall	8872	77.10	70.03	8768	111.16	51.51	8809	106.72	53.17	26,449	98.26	59.05
lnCH_4_												
Heifer 1	2985	4.28	14.85	2992	4.78	9.68	2985	4.77	8.93	8962	4.61	12.29
Heifer 2	2982	4.46	12.25	2980	4.48	12.58	2997	4.70	10.40	8959	4.55	11.98
Heifer 3	2962	3.79	14.68	2995	4.63	10.18	2978	4.22	14.41	8935	4.21	15.35
Overall	8929	4.18	15.48	8967	4.63	11.15	8960	4.56	12.47	26,856	4.46	13.76

**Table 3:** Number of records, aggregate value, slope, and coefficient of determination (R^2^) of the linear model calculated within the day on five measurements for methane (CH_4_) and log_e_-transformed methane (lnCH_4_) emissions.

Animals	Day 1				Day 2				Day 3			
	Records	Aggregate	Slope	R^2^	Records	Aggregate	Slope	R^2^	Records	Aggregate	Slope	R^2^
CH_4_, ppm × m												
Heifer 1	2941	248,837	83.16	0.991	2897	363,110	129.77	0.997	2913	360,499	127.42	0.997
Heifer 2	2943	281,808	101.84	0.997	2925	286,357	99.63	0.989	2945	349,011	125.67	0.996
Heifer 3	2988	153,393	52.06	0.997	2946	325,148	111.60	0.997	2951	230,607	80.08	0.996
lnCH_4_												
Heifer 1	2985	12,787	4.26	0.999	2992	14,313	4.82	0.999	2985	14,250	4.80	0.999
Heifer 2	2982	13,289	4.52	0.999	2980	13,354	4.49	0.999	2997	14,082	4.77	0.999
Heifer 3	2962	11,212	3.79	0.999	2995	13,858	4.64	0.999	2978	12,562	4.26	0.999

**Table 4:** Number of records, mean, repeatability relative standard deviation (RSD_r_, %) calculated within the day on five measurements, and reproducibility relative standard deviation (RSD_R_, %) calculated across days on 15 measurements for peaks of methane (CH_4_) and log_e_-transformed methane (lnCH_4_) emissions.

Item	Day 1			Day 2			Day 3			Overall		
	Records	Mean	RSD_r_	Records	Mean	RSD_r_	Records	Mean	RSD_r_	Records	Mean	RSD_R_
CH_4_, ppm × m												
Heifer 1	223	235.89	18.79	468	231.16	19.88	427	223.29	17.58	1118	229.10	18.95
Heifer 2	251	233.31	20.15	299	226.91	19.22	374	226.72	18.82	924	228.57	19.36
Heifer 3	67	224.42	18.84	342	233.79	19.37	194	229.71	19.22	603	231.44	19.29
Overall	541	233.28	19.47	1109	230.83	19.57	995	225.84	18.40	2645	229.45	19.17
lnCH_4_												
Heifer 1	243	5.60	5.13	484	5.58	4.90	424	5.54	4.87	1151	5.57	4.96
Heifer 2	253	5.59	4.50	312	5.57	5.34	357	5.54	4.78	922	5.56	4.91
Heifer 3	62	5.54	4.60	345	5.56	4.53	206	5.59	5.08	613	5.57	4.74
Overall	558	5.59	4.80	1141	5.57	4.91	987	5.55	4.89	2686	5.57	4.89
